# Distinct Influence of Hypercaloric Diets Predominant with Fat or Fat and Sucrose on Adipose Tissue and Liver Inflammation in Mice

**DOI:** 10.3390/molecules25194369

**Published:** 2020-09-23

**Authors:** Caíque S. M. Fonseca, Joshua E. Basford, David G. Kuhel, Eddy S. Konaniah, James G. Cash, Vera L. M. Lima, David Y. Hui

**Affiliations:** 1 Department of Pathology, Metabolic Diseases Research Center, University of Cincinnati College of Medicine, Cincinnati, OH 45237, USA; caiquesmfonseca@gmail.com (C.S.M.F.); basfordje@gmail.com (J.E.B.); kuheld@yahoo.com (D.G.K.); ed.konaniah@gmail.com (E.S.K.); cashjg@gmail.com (J.G.C.); 2 Grupo de Pesquisa em Doenças Metabólicas, Faculdade Tiradentes de Jaboatão dos Guararapes, Sociedade de Educação Tiradentes, Jaboatão dos Guararapes, Pernambuco 54410-100, Brazil; 3 Laboratório de Lipídeos, Departamento de Bioquímica, Centro de Biociências, Universidade Federal de Pernambuco, Recife, Pernambuco 50670-901, Brazil; lima.vera.ufpe@gmail.com

**Keywords:** dietary fat, sucrose, adipose tissue inflammation, NAFLD, steatohepatitis, metabolic disease, lipids

## Abstract

Overfeeding of a hypercaloric diet leads to obesity, diabetes, chronic inflammation, and fatty liver disease. Although limiting fat or carbohydrate intake is the cornerstone for obesity management, whether lowering fat or reducing carbohydrate intake is more effective for health management remains controversial. This study used murine models to determine how dietary fat and carbohydrates may influence metabolic disease manifestation. Age-matched C57BL/6J mice were fed 2 hypercaloric diets with similar caloric content, one with very high fat and low carbohydrate content (VHF) and the other with moderately high fat levels with high sucrose content (HFHS) for 12 weeks. Both groups gained more weight and displayed hypercholesterolemia, hyperglycemia, hyperinsulinemia, and liver steatosis compared to mice fed a normal low-fat (LF) diet. Interestingly, the VHF-fed mice showed a more robust adipose tissue inflammation compared to HFHS-fed mice, whereas HFHS-fed mice showed liver fibrosis and inflammation that was not observed in VHF-fed mice. Taken together, these results indicate macronutrient-specific tissue inflammation with excess dietary fat provoking adipose tissue inflammation, whereas moderately high dietary fat with extra sucrose is necessary and sufficient for hepatosteatosis advancement to steatohepatitis. Hence, liver and adipose tissues respond to dietary fat and sucrose in opposite manners, yet both macronutrients are contributing factors to metabolic diseases.

## 1. Introduction

Chronic overfeeding of a hypercaloric diet that exceeds energy expenditure and storage can cause metabolic imbalance that ultimately leads to cellular and tissue dysfunction and disease. Worldwide, it is estimated that there are 1.46 billion overweight adults and more than 500 million obese individuals [1]. Such pandemic is associated with several comorbidities, having a crucial causative role in insulin resistance, diabetes mellitus, metabolic syndrome, and non-alcoholic liver disease (NAFLD) [2,3]. Obesity is associated with expansion of the metabolically active adipose tissue leading to chronic low-grade inflammation and insulin resistance, which in turn can affect hepatic metabolism, leading to hepatic injury [4,5]. Interestingly, while obesity is a major risk factor for NAFLD [6], increasing evidence shows a significant prevalence of NAFLD in nonobese individuals [7]. Nevertheless, both obesity and NAFLD are characterized by excess lipid accumulation in ectopic tissues and are key components of the metabolic syndrome. Given the interplay between excess tissue lipid accumulation and metabolic disease manifestation, an integrated approach to identify the etiology of these chronic inflammatory diseases is of great interest.

Non-alcoholic fatty liver disease is the most common cause of liver dysfunction, affecting 10–30% of the general population globally [8,9]. The spectrum of NAFLD ranges from simple benign hepatic steatosis to nonalcoholic steatohepatitis (NASH), fibrosis, cirrhosis, and hepatocellular carcinoma [10]. Together with obesity and type 2 diabetes, the prevalence of NAFLD is rapidly increasing and is present in up to 85% of extremely obese individuals [11]. Despite the clear relationship among central obesity, insulin resistance, and NAFLD, the etiology and progression of NAFLD to NASH remains elusive. While lipid accumulation in hepatocytes is one prerequisite of NAFLD, the progression to NASH requires additional stressors. The current paradigm indicates that lipotoxicity and inflammation caused by specific lipid molecules such as nonesterified fatty acids, cholesterol, and ceramide are potential causative factors that accelerate NAFLD progression to NASH [12]. Additional evidence suggests that excess fructose may also have detrimental effects on NAFLD, NASH, and diabetes [13].

The initial phase of metabolic disease management is lifestyle modifications that include increasing physical activity and reducing excess calorie intake. In particular, reducing dietary intake of high energy density macronutrients is the cornerstone of many weight management programs [14]. In recent years, limiting fat or carbohydrate intake has been suggested as a key component not only for obesity management but also for metabolic health [15,16,17]. However, whether lowering fat or reducing carbohydrate intake is more effective remains controversial [18,19]. Several clinical trials have shown that low carbohydrate diets are more effective than low-fat diets in weight management [20,21,22]. However, low carbohydrate diets have also been shown to increase LDL-cholesterol levels, thus raising concerns about cardiovascular risk [21]. Additional studies revealed that the type of carbohydrate may be an important determinant of health outcome. In particular, substituting saturated fat with carbohydrates that have a low glycemic index has been shown to reduce the risk of cardiovascular disease, whereas substituting fat with carbohydrates that have a high glycemic index is associated with higher cardiovascular risk [23]. In contrast to these studies showing the effectiveness of dietary carbohydrate reduction for metabolic disease management, several other clinical studies showed that limiting caloric intake with different macronutrient recommendations, regardless of whether dietary fat or carbohydrates reduction, all results in similar weight loss [16,24]. Interestingly, a more recent study revealed that weight loss response to diets low in fat or carbohydrates can be predicted based on fasting plasma glucose and insulin levels [25]. These observations suggested that dietary fat and carbohydrates may influence metabolic disease manifestation through distinct mechanisms. This study was undertaken to compare the impact of a very high fat (VHF) diet with a moderately high-fat diet supplemented with excess sucrose (HFHS) on metabolic disease manifestation and to delineate how dietary fat and sucrose contribute to the disease process.

## 2. Results

### 2.1. Diet Effects on Food Intake, Body Weight, and Adiposity

**Figure 1:**
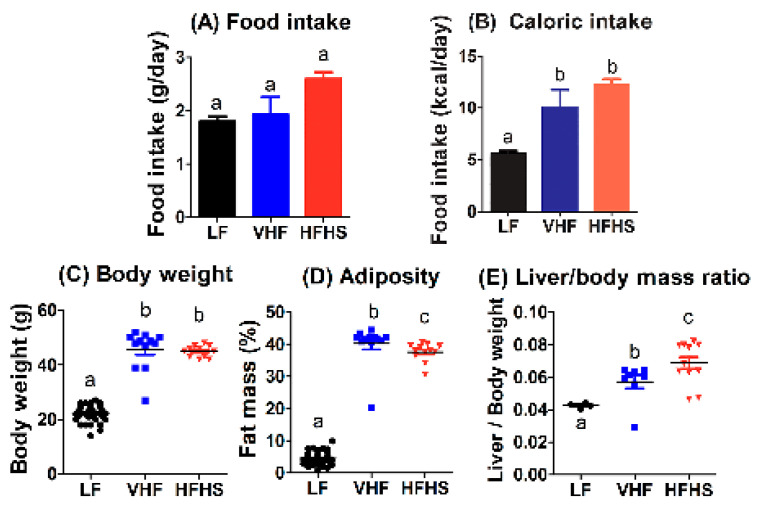
Diet effects on food intake, body weight, and adiposity. Male C57BL/6J mice were fed low fat (LF), very high fat (VHF), or moderately high fat with excess sucrose (HFHS) diets for 12 weeks. Food intake was determined over a 7-day period and the data were reported as grams of food consumed per day (**A**) or calories consumed per day (**B**). Body weight (**C**), adiposity determined based on fat mass as a percentage of body weight (**D**), and liver mass to body mass ratio (**E**) were measured after 12 weeks. The reported data represent the mean ± standard error from 49 LF-fed mice and 12 VHF-fed and 12 HFHS-fed mice. Data groups with letters a are significantly different from data groups with letters b or c at *p* < 0.01 (1-way ANOVA test with Bonferroni post hoc analysis). Data groups with letters b and c are significantly different at *p* < 0.001 by Mann–Whitney test.

Male age- and weight-matched C57BL/6J mice were fed VHF or HFHS diets with similar caloric content for 12 weeks prior to metabolic phenotype comparison with mice fed the low-fat (LF) diet. All mice consumed similar amounts of food during this period (Figure 1A), resulting in increased calorie intake by VHF- and HFHS-fed mice compared to LF-fed mice (Figure 1B). Mice fed the VHF and HFHS diets displayed significant body weight gain and adiposity compared to LF-fed mice, but no difference in body weight was observed between VHF- and HFHS-fed mice (Figure 1C). Interestingly, significantly less adiposity, as determined by fat mass, was observed in HFHS-fed mice compared to VHF-fed mice (Figure 1D). In contrast, liver weight was higher in HFHS-fed mice compared to VHF-fed mice (Figure 1E).

### 2.2. Diet Effects on Plasma Lipid and Lipoproteins

**Figure 2:**
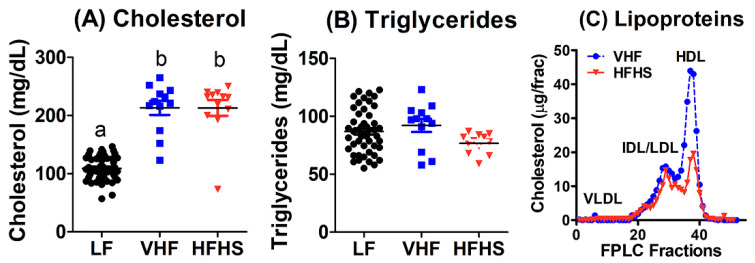
Diet effects on plasma lipid and lipoproteins. Male C57BL/6J mice were fed low fat (LF), very high fat (VHF), or moderately high fat with excess sucrose (HFHS) diets for 12 weeks. Fasting plasma cholesterol (**A**) and triglyceride (**B**) levels were determined from 49 LF-fed mice and 12 VHF-fed and 12 HFHS-fed mice. Data groups with letter a are significantly different from data groups with letter b at *p* < 0.01 (1-way ANOVA with Bonferroni post hoc analysis). (**C**) Representative plasma lipoprotein profile determined after fast-performance liquid chromatography (FPLC) separation of plasma samples from VHF- and HFHS-fed mice.

Analysis of fasting plasma lipid levels revealed that VHF and HFHS diets increased plasma cholesterol levels to similar extent, but their fasting plasma triglyceride levels were similar to that observed in LF-fed mice (Figure 2A,B). Previous studies have reported that plasma lipids in wild type C57BL/6J mice are mostly associated with HDL lipoprotein particles when fed low fat diets. When the mice were fed the VHF or HFHS diets, plasma cholesterol was found in both IDL/LDL and HDL fractions (Figure 2C).

### 2.3. Diet Effects on Fasting Glucose and Insulin Levels and Insulin Resistance

**Figure 3:**
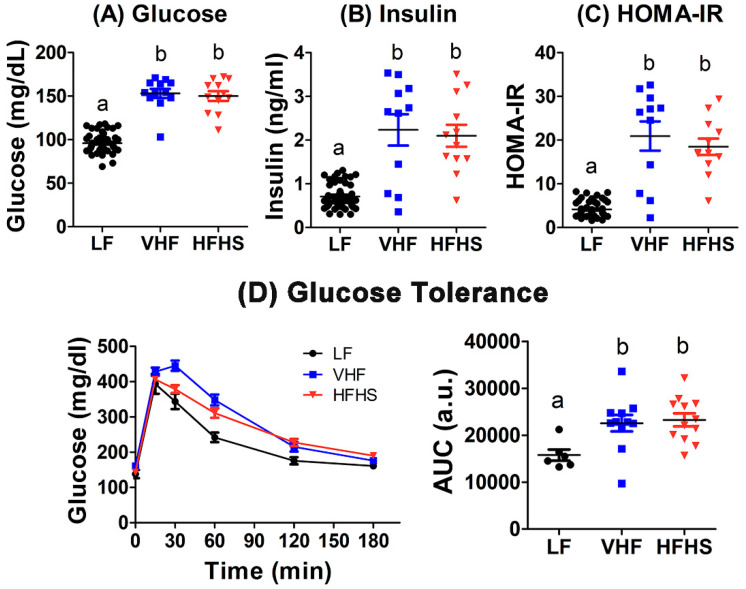
Diet effects on plasma glucose, insulin, and insulin resistance. Male C57BL/6J mice were fed low fat (LF), very high fat (VHF), or moderately high fat with excess sucrose (HFHS) diets for 12 weeks. Fasting blood glucose (**A**) and insulin (**B**) levels were determined from 49 LF-fed mice and 12 VHF-fed and 12 HFHS-fed mice. (**C**) The glucose and insulin data from each mouse were used to calculate HOMA index of insulin resistance (HOMA-IR). (**D**) a bolus glucose solution (2 g/kg body weight) was administered intraperitoneally to 6 LF-fed mice, 11 VHF-fed mice, and 12 HFHS-fed mice. Blood glucose levels were monitored over a 3 h period to determine glucose tolerance. Area under the curve (AUC) analysis of the data was used for statistical evaluation. Data groups with letter a are significantly different from data groups with letters b at *p* = 0.01 (1-way ANOVA with Bonferroni post hoc analysis).

Mice fed the VHF or HFHS diets displayed elevated fasting plasma glucose and insulin levels compared to LF-fed mice (Figure 3A,B), leading to higher HOMA index of insulin resistance (HOMA-IR) index suggestive of insulin resistance after VHF or HFHS feeding (Figure 3C). Indeed, when glucose tolerance test was administered to these animals by intraperitoneal glucose injection after an overnight fast, slower plasma glucose clearance was observed in both VHF- and HFHS-fed mice (Figure 3D). Area under the curve analysis showed that both VHF and HFHS diets reduced glucose tolerance to similar extent (Figure 3D). Since male C57BL/6J mice are resistant to intravenous glucose-induced insulin secretion [26], the slower plasma glucose clearance observed in VHF- and HFHS-fed mice is consistent with insulin resistance conferred by these diets.

### 2.4. Hypercaloric Diet with Excess Fat Promotes Adipose Tissue Inflammation

**Figure 4:**
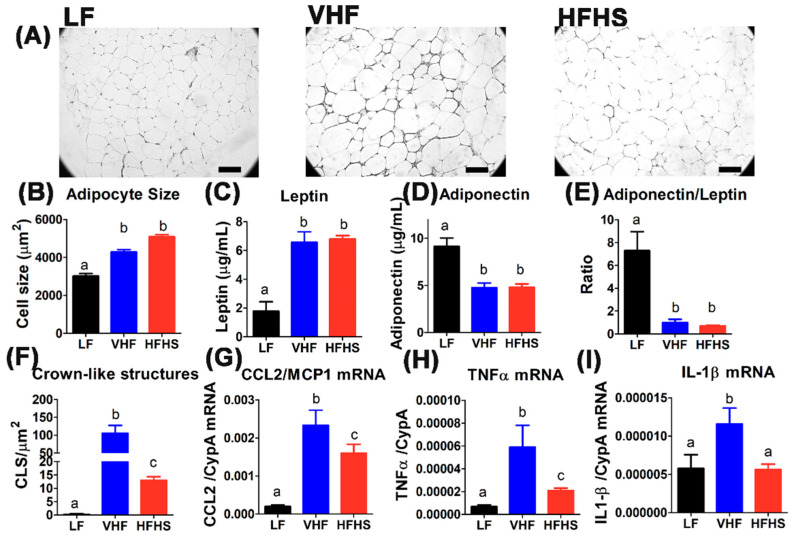
Hypercaloric diet with excess fat promotes adipose tissue inflammation. Epididymal adipose tissues were collected from C57BL/6J mice fed low fat (LF), very high fat (VHF), or moderately high fat with excess sucrose (HFHS) diets for 12 weeks. Representative adipose tissue section images are shown in (**A**). (**B**) Adipocyte size was determined by measuring the size of 160 adipocytes from 10 LF-fed mice and 475 adipocytes from 12 VHF-fed and 12 HFHS-fed mice. Data groups with letter a are statistically different from data groups with letter b at *p* < 0.01 (1-way ANOVA with Bonferroni post hoc analysis). Plasma leptin (**C**) and adiponectin (**D**) levels were determined from 10 LF-fed mice, 14 VHF-fed mice, and 17 HFHS-fed mice. The data were used to determine adiponectin/leptin ratio (**E**). Number of crown-like structures detected in adipose tissues sections of 10 LF-fed, 12 VHF-fed, and 12 HFHS-fed mice is shown in (**F**). Data groups with letter a are significantly different from data groups with letter b at *p* < 0.01 (1-way ANOVA with Bonferroni post hoc analysis). RNA extracted from adipose tissues of 7 mice in each group to measure expression levels of CCL2/monocyte chemoattractant protein-1 (MCP1) (**G**), tumor necrotic factor-α (TNFα) (**H**), and interleukin-1β (IL-1β) (**I**). The gene expression data with different letters indicate statistically significant difference at *p* < 0.05 (1-way ANOVA with Bonferroni post hoc analysis).

To determine changes in adipose tissue architecture that may occur after feeding the experimental diets, epididymal adipose tissues were harvested from the animals at the time of sacrifice. Histological analysis of the adipose tissues revealed that both VHF and HFHS diet feeding increased adipocyte size indicative of adipocyte hypertrophy (Figure 4A,B). The increased adipocyte cell size upon VHF and HFHS diet feedings was reflected by elevated leptin levels in plasma (Figure 4C). In contrast, plasma adiponectin levels were significantly lower in VHF- and HFHS-fed mice compared to LF-fed mice (Figure 4D), leading to a robust decrease in adiponectin-to-leptin ratio indicative of adipocyte dysfunction and inflammation (Figure 4E). Increased adipocyte dysfunction and adipose tissue inflammation in VHF- and HFHS-fed mice was also evident by observation of increased number of crown-like structures (Figure 4F) that denote dead adipocytes surrounded by macrophages [27]. The adipose tissues in VHF- and HFHS-fed mice also displayed increased expression of monocyte chemoattractant protein-1 (MCP1, also known as CCL2) (Figure 4G), as well as inflammatory cytokines such as tumor necrotic factor-α (TNFα) and interleukin-1β (IL-1β) (Figure 4H,I). Interestingly, while crown-like structures and inflammatory cytokines were evident in adipose tissues of HFHS-fed mice but not in adipose tissues of LF-fed mice, a more robust increase in crown-like structures and inflammatory cytokines were observed in adipose tissues of VHF-fed mice compared to LF-fed as well as HFHS-fed mice (Figure 4F–I). Taken together, these results indicate that excess fat in the diet provoked adipocyte dysfunction and inflammation more robustly than a diet with similar caloric content but contained lower amount of fat and high amount of sucrose.

### 2.5. High-Fat High-Sucrose Diet Accelerates Hepatosteatosis Transition to Steatohepatitis

**Figure 5:**
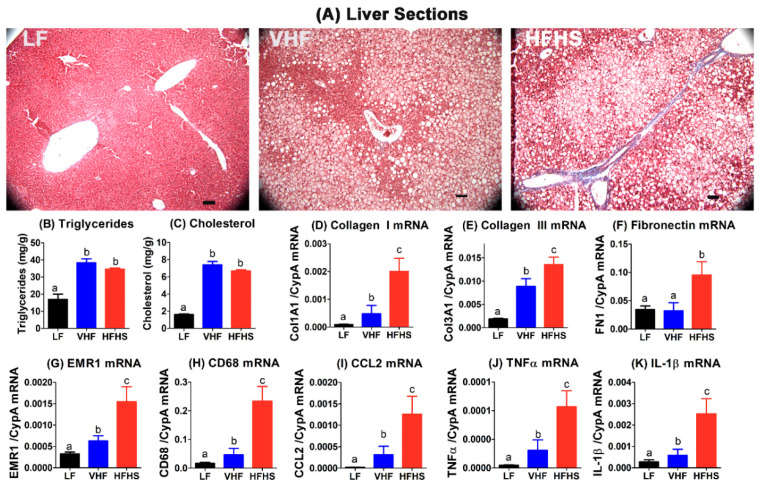
High-fat diet with excess sucrose accelerates hepatosteatosis transition to steatohepatitis. Livers were collected from C57BL/6J mice fed low fat (LF), very high fat (VHF), or moderately high fat with excess sucrose (HFHS) diets for 12 weeks. Representative liver tissue section images are shown in (**A**). Scale bar = 50 µm. Liver triglyceride (**B**) and cholesterol content (**C**) were determined from 6 mice in each group. Bars in groups with letters a and b are significantly different at *p* < 0.05 (1-way ANOVA with Bonnferroni post hoc analysis). RNA was extracted from the livers of 5 LF-fed mice, 7 VHF-fed mice, and 9 HFHS-fed mice to measure expression levels of collagen-I (**D**), collagen-III (**E**), fibronectin (**F**), EMRI (F4/80) (**G**), CD68 (**H**), CCL2/MCP1 (**I**), TNFα (**J**), and IL-1β (**K**). In the gene expression data, bars with different letters indicate statistically significant difference between groups at *p* < 0.05 (1-way ANOVA with Bonferroni post hoc analysis).

In comparison to LF-fed mice, the livers isolated from both VHF-fed and HFHS-fed mice displayed more lipid droplets and elevated triglyceride and cholesterol levels consistent with hepatosteatosis (Figure 5A–C). Interestingly, Masson’s trichrome staining of the tissue sections showed only sporadic fibrotic bridges in the livers of VHF-fed mice. In contrast, a 2–3-fold increase in fibrotic bridges was observed in the livers of HFHS-fed mice (Figure 5A). The increased fibrosis observed in HFHS-fed mice may account for the increased liver mass observed in these animals. These histological data were consistent with higher collagen I and collagen III expression levels in the livers of HFHS-fed mice compared to those observed in VHF-fed mice, which were also higher than those observed in LF-fed mice (Figure 5D,E). Additionally, increased fibronectin gene expression was observed only in the livers of HFHS-fed mice but not in VHF-fed mice (Figure 5F). Moreover, in addition to increased expression of genes related to fibrosis, increased expression of macrophage markers EMRI1 (F4/80) and CD68 as well as inflammatory cytokines such as MCP1/CCL2, TNFα, and IL-1β was also observed in the livers of VHF- and HFHS-fed mice compared to LF-fed mice (Figure 5G–K). Interestingly, expression of macrophage markers and inflammatory cytokines was also much higher in the livers of HFHS-fed mice compared to VHF-fed mice (Figure 5G–K). Taken together, these data indicate that in contrast to adipose tissues, a high sucrose content in the diet is necessary to accelerate liver fibrosis and inflammation, and a similar hypercaloric diet with only high fat but low sucrose content only caused hepatosteatosis with minimal progression to steatohepatitis.

## 3. Discussion

Numerous human population studies and experiments with various animal models have clearly documented the contributory influence of high caloric intake on metabolic diseases. In murine models, various diets have been used in different laboratories to instigate obesity, diabetes, and fatty liver disease. The most popular diets among the laboratories include the high-fat diet-induced obesity (DIO) diet that contains 5.21 kcal/gm with 60% of calories derived from fat and 6.8% calories derived from sucrose, the Western diet that contains 4.7 kcal/gm with 41% calories derived from fat and 29% calories derived from sucrose along with 0.2% cholesterol, and a diabetogenic diet that contains 5.56% kcal/gm with 58% calories derived from fat and 12.5% calories derived from sucrose. Although studies invariably showed that rodents consuming these diets all displayed increased body weight, insulin resistance, and hepatosteatosis, and diets containing excess cholesterol or sucrose also accelerates hepatosteatosis transition to steatohepatitis, the different amounts of calories in each diet precludes a definitive conclusion regarding the impact of specific macronutrients in metabolic disease manifestation. Experiments conducted in various laboratories are also difficult to compare due to differences in housing environments that may have direct influence on metabolic diseases [28,29].

The goal of this study is to use male C57BL/6J mice housed under the same institutional environment to compare the influence of a very high-fat diet (60 kcal%) with low carbohydrate (20% kcal%; 6.8 kcal% derived from sucrose) content versus a similar hypercaloric diet with lower fat content (44.6 kcal%) but enriched with sucrose (40.7 kcal% carbohydrate) on metabolic disease and tissue inflammation. Results showed that both VHF and HFHS diets promote body weight gain, but the HFHS-fed mice displayed slightly less adiposity than VHF-fed mice. Nevertheless, both VHF and HFHS feeding promote hypercholesterolemia, hyperglycemia, hyperinsulinemia, and insulin resistance to a similar extent. Both VHF and HFHS diets also caused adipocyte dysfunction to a similar extent as indicated by a similar increase in leptin levels and reduced adiponectin levels in plasma. Leptin is an adipokine that promotes utilization of fatty acids instead of glucose as the fuel source in peripheral tissues by stimulating lipolysis without increasing fatty acid release [30]. However, excess lipolysis without concomitant fatty acid release ultimately leads to lipotoxicity and the release of proinflammatory cytokines [31,32]. In contrast, adiponectin is an adipokine that suppresses inflammatory cytokine expression [33]. Hence, the reduced adiponectin to leptin ratio observed in plasma of VHF- and HFHS-fed mice compared to LF-fed mice can be interpreted as adipocyte dysfunction caused by VHF and HFHS diets. Interestingly, while increased adipose tissue inflammation was observed in both VHF- and HFHS-fed mice compared to LF-fed mice, adipose tissue inflammation was significantly more robust by feeding the VHF diet compared to HFHS diet. In contrast, while both VHF and HFHS diets caused similar liver lipid accumulation, liver fibrosis and inflammation were more robust in HFHS-fed mice compared to VHF-fed mice. Taken together, these results indicate macronutrient-specific tissue inflammation with excess dietary fat provoking adipose tissue inflammation, whereas a moderately high dietary fat diet with extra dietary sucrose is necessary and sufficient for hepatosteatosis advancement to steatohepatitis. Thus, metabolic diseases induced by these diets are likely the combined effects of inflammation in adipose tissue and the liver [34]. 

The preferential influence of VHF diet on adipose tissue inflammation instead of liver inflammation is consistent with results reported previously [35]. Other studies have also indicated that a hypercaloric diet with fat as the predominant energy-rich macronutrient provokes steatosis in the liver but does not cause liver inflammation and fibrosis that are typically observed in steatohepatitis [36]. Thus, while steatosis is a prerequisite of hepatosteatosis, a second hit due to environmental factors or genetic variations is necessary for liver disease progression to steatohepatitis. Several studies have shown that excessive dietary cholesterol or fructose is necessary to complement the excessive fat in the diet for NAFLD progression to NASH [37,38,39]. However, these studies were performed by comparing the impact of high fat diet with or without cholesterol or sucrose/fructose supplementation in liver disease progression. Thus, whether diets with similar caloric content but with different high energy macronutrient composition are similar in liver disease progression needs to be clarified. Moreover, whether a high sucrose diet with moderately high fat content also promotes adipose tissue inflammation is unknown. Results of the current study provided conclusive evidence that excess sucrose added to a moderately high fat diet is sufficient to instigate liver inflammation and fibrosis, thus documenting the adverse impact of excessive sucrose in liver health. The contributory role of excess sucrose on liver diseases is likely due to the fructose moiety in sucrose, which can be rapidly metabolized in the liver by the liver-specific fructokinase C to generate substrates for de novo lipogenesis and leads to increased uric acid levels [40]. 

Our study showed the surprising results that substituting some of the excess fat with sucrose actually reduced adipose tissue inflammation. We speculate that the liver-specific sucrose/fructose metabolism may result in less availability of this dietary nutrient to adipose tissues, hence reducing adipocyte nutrient overload and limiting adipose tissue inflammation. While this hypothesis remains tentative and requires more rigorous experimentation, the observations that HFHS-fed mice displayed less crown-like structures and inflammatory macrophages in adipose tissues are consistent with this hypothesis. Interestingly, our results are in striking contrast to a recent report that dietary sucrose induces metabolic inflammation, including inflammation in adipose tissues, more than dietary fat in *Ldlr^−/−^ApoB^100/100^* mice [41]. However, there are key differences between the 2 studies that led to the disparate conclusions. First, our study compared dietary fat and sucrose effects using a diet with very high fat and low sucrose content and a diet with moderately high fat content supplemented with excess sucrose. In contrast, the study with *Ldlr^−/−^ApoB^100/100^* mice compared diets with a high fat low sucrose content with one that contains low fat and high sucrose. More importantly, the genetic background of the experimental animals may be an important determinant in how fat and sucrose influences liver and adipose tissue inflammation. In particular, in the absence of LDL receptor in the *Ldlr^−/−^ApoB^100/100^* mice, the impaired postprandial lipoprotein clearance by the liver may increase lipid partitioning to adipose tissues to promote adiposity and tissue inflammation at the expense of liver inflammation [42]. In contrast, lipoprotein uptake mediated by LDL receptor is expected to work synergistically with dietary sucrose to promote liver steatosis and inflammation.

Results showing differences in how dietary fat and sucrose impact tissue inflammation in wild type C57BL/6J mice and *Ldlr^−/−^ApoB^100/100^* mice highlight the importance of genetic factors in dictating tissue handling of dietary macronutrients. This conclusion is consistent with the report that weight loss and metabolic response to diets low in fat or carbohydrates can be predicted based on fasting plasma glucose and insulin levels [25]. Accordingly, genetic variations need to be taken into consideration for dietary advice to limit fat or carbohydrate intake for the management of obesity, diabetes, and fatty liver disease. Obviously, a more prudent diet low in both fat and carbohydrate is the optimal approach.

## 4. Materials and Methods

### 4.1. Animals

All experiments were conducted in accordance with the guidelines of the University of Cincinnati Institutional Animal Use and Care Committee (approval number: 17-09-08-01, expiration 14 November 2020) in accordance with the Guide for the Care and Use of Laboratory Animals of the National Institutes of Health. Age-matched (10-weeks-old) male C57BL/6 mice were housed with 1 to 4 mice per cage in a temperature- and light-controlled facility and had free access to food and water during the period of study. The mice were fed a low-fat rodent diet (LM485, Harlan-Teklad, Madison, WI, USA), very high-fat diet (VHF, Research Diets D12492), or high-fat high-sucrose diet (HFHS, Teklad TD08811) for 12 weeks for experiments. Composition of each diet as determined by the suppliers is shown in Table S1. Body weight was measured using a Denver 300K scale and adiposity was determined as fat mass by echo magnetic resonance imaging using a Whole-Body Composition Analyzer (Echo Medical, Houston, TX, USA).

### 4.2. Analytical Procedures

Blood was collected from conscious mice without anesthetics after an overnight fast. Blood glucose levels were measured using an Accu-Check Active Glucometer (Roche Applied Science, Indianapolis, IN, USA). Plasma insulin (Crystal Chem, Chicago, IL, USA), leptin (R&D Systems, Minneapolis, MN, USA), and adiponectin (R&D Systems, Minneapolis, MN, USA) were measured by ELISA kits. Plasma and liver cholesterol and triglyceride levels were determined using Affinity cholesterol and triglyceride kits (Thermo Fisher Scientific, Waltham, MA, USA). For lipoprotein separation, 100 µL of plasma was subjected to fast-performance liquid chromatography (FPLC) on 2 Superose 6 HR 10/30 columns connected in series (Amersham Biosciences, Piscataway, NJ, USA), and 0.5 mL fractions were collected for cholesterol determinations. Fractions identified as VLDL, IDL/LDL, and HDL were based on elution profile of standard lipoproteins. For liver lipid measurements, fasted mice were anesthetized by isoflurane exposure prior to sacrifice for tissue harvesting. Liver lipids were then extracted from the tissues for cholesterol and triglyceride determinations as described [43].

### 4.3. Glucose Tolerance Test

Mice were fasted overnight prior to receiving a glucose solution (2 g/kg body weight) by intraperitoneal injection. Blood glucose levels were measured from conscious mice without anesthetics over a 3 h period. Rate of glucose excursion was determined by area under the curve analysis of the data.

### 4.4. Tissue Histology

Liver and epididymal adipose tissues were excised from isoflurane-anesthetized animals after perfusion with 3 mL of 10% formalin in phosphate-buffered saline. The tissues were fixed in isotonic neutral 4% paraformaldehyde for 3 days, embedded in paraffin, and then cross-sectioned. Sections from both organs were stained with hematoxylin and eosin. Liver sections were also stained with Masson’s Trichrome stain. Crown-like structures were identified by the presence of nucleated cells surrounding individual adipocytes. Each stain was carried out on at least four mice per group and the images in the figures are representative of each group.

### 4.5. Real-Time Quantitative Polymerase Chain Reaction Analysis

Liver and epididymal adipose tissues were collected from isoflurane-anesthetized animals, snap-frozen, and stored at −80°C until analysis. Total RNA (*n* = 5 to 7 per group) was extracted with TRIzol reagent (Invitrogen) followed by a further isolation using RNeasy plus mini kit (Qiagen, Germantown, MD, USA) and treatment with Turbo DNase (Applied Biosystems/Ambiom, Austin, TX, USA). cDNA was made using iScript cDNA synthesis kit (Bio-Rad Laboratories, Hercules, CA, USA). Real-time quantitative PCR was performed using a StepOnePlus Fast Thermocycler using Fast SYBR Green Mix (Applied Biosystems, Carlsbad, CA, USA) with primer sequences as shown in Table S2. Gene expression values were normalized to the value of the housekeeping gene CycA (Cyclophilin A) and calculated based on the comparative cycle threshold Ct method (2^−ΔΔCt^).

### 4.6. Statistical Analysis

Data analysis was performed using GraphPad Prism 5 (GraphPad Software Inc., La Jolla, CA, USA) and are presented as mean ± standard error. Analysis of Variance (ANOVA) followed by Bonferroni multiple comparison post hoc test was used to detect differences among groups that had normal distribution. A *p*-value < 0.05 was considered statistically significant and indicated by different letters in the bar graphs.

## 5. Conclusions

In conclusion, results of this study showed macronutrient-specific tissue inflammation with excess dietary fat without extra sucrose provoking adipose tissue inflammation, whereas excess dietary fat with extra sucrose is necessary for hepatosteatosis advancement to steatohepatitis. Nevertheless, both VHF and HFHS diets caused hypercholesterolemia, hyperglycemia, and hyperinsulinemia to a similar extent. The implication of this study is that a more prudent diet low in both fat and carbohydrate is the optimal approach for metabolic disease management.

## Supplementary Materials

The following are available online, Table S1: Macronutrient composition of the diets; Table S2: Primer sequences used for RT-PCR amplification of RNA.

## References

[1] Finucane M.M., Stevens G.A., Cowan M.J., Danaei G., Lin J.K., Paciorek C.J., Singh G.M., Gutierrez H.R., Lu Y., Bahalim A.N. (2011). National, regional, and global trends in body-mass index since 1980: Systematic analysis of health examination surveys and epidemiological studies with 960 country-years and 9·1 million participants. Lancet.

[2] Nguyen N.T., Magno C.P., Lane K.T., Hinojosa M.W., Lane J.S. (2008). Association of hypertension, diabetes, dyslipidemia, and metabolic syndrome with obesity: Findings from the National Health and Nutrition Examination Survey, 1999 to 2004. J. Am. Coll. Surg..

[3] Corey K.E., Kaplan L.M. (2014). Obesity and liver disease. The epidemic of the twenty-first century. Clin. Liver Dis..

[4] Bertola A., Bonnafous S., Anty R., Patouraux S., Saint-Paul M.C., Iannelli A., Gugenheim J., Barr J., Mato J.M., Le Marchand-Brustel Y. (2010). Hepatic expression patterns of inflammatory and immune response genes associated with obesity and NASH in morbidly obese patients. PLoS ONE.

[5] Cummins T.D., Holden C.R., Sansbury B.E., Gibb A.A., Shah J., Zafar N., Tang Y., Hellmann J., Rai S.N., Spite M. (2014). Metabolic remodeling of white adipose tissue in obesity. Am. J. Physiol. Endocrinol. Metab..

[6] Li L., Liu D.W., Yan H.Y., Wang Z.Y., Zhao S.H., Wang B. (2016). Obesity is an independent risk factor for non-alcoholic fatty liver disease: Evidence from a meta-analysis of 21 cohort studies. Obes. Rev..

[7] Aby E., Saab S. (2017). Nonobese nonalcoholic fatty liver disease. Clin. Liver Dis..

[8] Lazo M., Clark J.M. (2008). The epidemiology of nonalcoholic fatty liver disease: A global perspective. Semin. Liver Dis..

[9] Vernon G., Baranova A., Younossi Z.M. (2011). Systematic review: The epidemiology and natural history of non-alcoholic fatty liver disease and non-alcoholic steatohepatitis in adults. Aliment. Pharmacol. Ther..

[10] Farrell G.C., van Rooyen D., Gan L., Chitturi S. (2012). NASH is an inflammatory disorder: Pathogenic, prognostic and therapeutic implications. Gut Liver.

[11] Gholam P.M., Kotler D.P., Flancbaum L.J. (2002). Liver pathology in morbidly obese patients undergoing Roux-en-Y gastric bypass surgery. Obes. Surg..

[12] Neuschwander-Tetri B.A. (2010). Hepatic lipotoxicity and the pathogenesis of nonalcoholic steatohepatitis: The central role of nontriglyceride fatty acid metabolites. Hepatology.

[13] Mai B.H., Yan L.-J. (2019). The negative and detrimental effects of high fructose on the liver, with special reference to metabolic disorders. Diabetes Metab. Syndr. Obes..

[14] Smethers A.D., Rolls B.J. (2018). Dietary management of obesity: Cornerstones of healthy eating patterns. Med. Clin. N. Am..

[15] Hooper L., Abdelhamid A., Moore H.J., Douthwaite W., Skeaff C.M., Summerbell C.D. (2012). Effect of reducing total fat intake on body weight: Systematic review and meta-analysis of randomised controlled trials and cohort studies. BMJ.

[16] Sacks F.M., Bray G.A., Carey V.J., Smith S.R., Ryan D.H., Anton S.D., McManus K., Champagne C.M., Bishop L.M., Laranjo N. (2009). Comparison of weight-loss diets with different compositions of fat, protein, and carbohydrates. N. Engl. J. Med..

[17] Fogelholm M., Anderssen S., Gunnarsdottir I., Lahti-Koski M. (2012). Dietary macronutrients and food consumption as determinants of long-term weight change in adult populations: A systematic literature review. Food Nutr. Res..

[18] Bray G.A., Siri-Tarino P.W. (2016). The role of macronutrient content in the diet for weight management. Endocrinol. Metab. Clin. N. Am..

[19] Makris A., Foster G.D. (2011). Dietary approaches to the treatment of obesity. Psychiatr. Clin. N. Am..

[20] Bueno N.B., de Melo I.S.V., de Oliveira S.L., da Rocha Ataide T. (2013). Very low carbohydrate ketogenic diet v. low fat diet for long term weight loss: A meta-analysis of randomised controlled trials. Br. J. Nutr.

[21] Mansoor N., Vinknes K.J., Veierod M.B., Retterstol K. (2016). Effects of low carbohydrate diets v. low fat diets on body weight and cardiovascular risk factors: A meta-analysis of randomised controlled trials. Br. J. Nutr..

[22] Sackner-Bernstein J., Kanter D., Kaul S. (2015). Dietary intervention for overweight and obese adults: Comparison of low carbohydrate and low fat diets. A meta-analysis. PLoS ONE.

[23] Jakobsen M.U., Dethlefsen C., Joensen A.M., Stegger J., Tjonneland A., Schmidt E.B., Overvad K. (2010). Intake of carbohydrates compared with intake of saturated fatty acids and risk of myocardial infarction: Importance of the glycemic index. Am. J. Clin. Nutr.

[24] Foster G.D., Wyatt H.R., Hill J.O., Makris A.P., Rosenbaum D.L., Brill C., Stein R.I., Mohammed B.S., Miller B., Rader D.J. (2010). Weight and metabolic outcomes after 2 years on a low-carbohydrate versus low-fat diet: A randomized trial. Ann. Intern. Med..

[25] Hjorth M.F., Astrup A., Zohar Y., Urban L.E., Sayer R.D., Patterson B.W., Herring S.J., Klein S., Zemel B.S., Foster G.D. (2019). Personalized nutrition: Pretreatment glucose metabolism determines individual long-term weight loss responsiveness in individuals with obesity on low carbohydrate versus low fat diet. Int. J. Obes..

[26] Fergusson G., Etheir M., Guevremont M., Chretien C., Attane C., Joly E., Fioramonti X., Prentki M., Poitout V., Alquier T. (2014). Defective insulin secretory response to intravenous glucose in C57BL/6J compared to C57BL/6N mice. Mol. Metab..

[27] Cinti S., Mitchell G., Barbatelli G., Murano I., Ceresi E., Faloia E., Wang S., Fortier M., Greenberg A.S., Obin M.S. (2005). Adipocyte death defines macrophage localization and function in adipose tissue of obese mice and humans. J. Lipid Res..

[28] Toth L.A. (2015). The influence of the cage environment on rodent physiology and behavior: Implications for reproducibility of pre-clinical rodent research. Exp. Neurol..

[29] Leulier F., MacNeil L.T., Lee W.-J., Rawls J.F., Cani P.D., Schwarzer M., Zhao L., Simpson S.J. (2017). Integrative physiology: At the crossroads of nutrition, microbiota, animal physiology, and human health. Cell Metab..

[30] Hynes G.R., Jones P.J.H. (2001). Leptin and its role in lipid metabolism. Curr. Opin. Lipidol..

[31] Lopez-Jaramillo P., Gomez-Arbelaez D., Lopez-Lopez J., Lopoz-Lopez C., Martinez-Ortega J., Gomez-Rodriguez A., Triana-Cubillos S. (2014). The role of leptin/adiponectin ratio in metabolic syndrome and diabetes. Horm. Mol. Biol. Clin. Investig..

[32] Iikuni N., Lam Q.L., Lu L., Matarese G., La Cava A. (2008). Leptin and inflammation. Curr. Immunol. Rev..

[33] Ouchi N., Walsh K. (2007). Adiponectin as an anti-inflammatory factor. Clin. Chim. Acta.

[34] Johnson A.M.F., Olefsky J.M. (2013). The origins and drivers of insulin resistance. Cell.

[35] Van der Heijden R.A., Sheedfar F., Morrison M.C., Hommelberg P.P.H., Kor D., Kloosterhuis N.J., Gruben N., Youssef S.A., de Bruin A., Hofker M.H. (2015). High fat diet induced obesity primes inflammation in adipose tissue prior to liver in C57BL/6J mice. Aging.

[36] Day C.P., James O.F. (1998). Steatohepatitis: A tale of two hits. Gastroenterology.

[37] Wouters K., van Gorp P.J., Bieghs V., Gijbels M.J., Duimel H., Lutjohann D., Kerksiek A., van Kruchten R., Maeda N., Staels B. (2008). Dietary cholesterol, rather than liver steatosis, leads to hepatic inflammation in hyperlipidemic mouse models of nonalcoholic steatohepatitis. Hepatology.

[38] Savard C., Tartaglione E.V., Kuver R., Haigh W.G., Farrell G.C., Subramanian S., Chait A., Yeh M.M., Quinn L.S., Ioannou G.N. (2013). Synergistic interaction of dietary cholesterol and dietary fat in inducing experimental steatohepatitis. Hepatology.

[39] Kohli R., Kirby M., Xanthakos S.A., Softic S., Feldstein A.E., Saxena V., Tang P.H., Miles L., Miles M.V., Balistreri W.F. (2010). High-fructose, medium chain trans fat diet induces liver fibrosis and elevates plasma coenzyme Q9 in a novel murine model of obesity and nonalcoholic steatohepatitis. Hepatology.

[40] Stanhope K.L., Schwarz J.-M., Havel P.J. (2013). Adverse metabolic effects of dietary fructose: Results from the recent epidemiological, clinical, and mechanistic studies. Curr. Opin. Lipidol..

[41] Perazza L.R., Mitchell P.L., Jensen B.A.H., Daniel N., Boyer M., Varin T.V., Bouchareb R., Nachbar R.T., Bouchard M., Blais M. (2020). Dietary sucrose induces metabolic inflammation and atherosclerotic cardiovascular diseases more than dietary fat in *Ldlr^−/−^ApoB^100/100^* mice. Atherosclerosis.

[42] Veniant M.M., Beigneux A.P., Bensadoun A., Fong L.G., Young S.G. (2008). Lipoprotein size and susceptibility to atherosclerosis—Insights from genetically modified mouse models. Curr. Drug Targets.

[43] Alogaili F., Chinnarasu S., Jaeschke A., Kranias E.G., Hui D.Y. (2020). Hepatic HAX-1 inactivation prevents metabolic diseases by enhancing mitochondrial activity and bile salt export. J. Biol. Chem..

