# Supplementary material for: Distinct Influence of Hypercaloric Diets Predominant with Fat or Fat and Sucrose on Adipose Tissue and Liver Inflammation in Mice
Source: Molecules. 2020 Sep 23;25(19):4369. doi: 10.3390/molecules25194369 (PMC7584000; doi:10.3390/molecules25194369)
Supplement: Supplementary file 1 [file molecules-25-04369-s001.pdf]

Communication

Distinct Influence of Hypercaloric Diets Predominant with Fat or Fat and Sucrose on Adipose Tissue and Liver Inflammation in Mice

Caíque S. M. Fonseca [1,2], Joshua E. Basford [1], David G. Kuhel [1], Eddy S. Konaniah [1], James G. Cash [1], Vera L. M. Lima [3] and David Y. Hui [1,*]

1 Department of Pathology, Metabolic Diseases Research Center, University of Cincinnati College of Medicine, Cincinnati, OH 45237, USA; caiquesmfonseca@gmail.com (C.S.M.F.); basfordje@gmail.com (J.E.B.); kuheld@yahoo.com (D.G.K.); ed.konaniah@gmail.com (E.S.K.); cashjg@gmail.com (J.G.C.)

2 Grupo de Pesquisa em Doenças Metabólicas, Faculdade Tiradentes de Jaboatão dos Guararapes, Sociedade de Educação Tiradentes, Jaboatão dos Guararapes, Pernambuco, 54410-100, Brazil

3 Laboratório de Lipídeos, Departamento de Bioquímica, Centro de Biociências, Universidade Federal de Pernambuco, Recife, Pernambuco, 50670-901, Brazil; lima.vera.ufpe@gmail.com

* Correspondence: huidy@ucmail.uc.edu; Tel.: +01-513-558-9152

Table S1. Macronutrient composition of diets

**Macronutrients**	LF (Teklad LM-485)		VHF (Research Diets D12492)		HFHS (Teklad TD08811)	
	% by weight	% kcal	% by weight	% kcal	% by weight	% kcal
Protein	19.1	25	26.0	20	17.3	14.7
Fat	5.8	17	35.0	60	23.2	44.6
Carbohydrate	44.3	58	26.0	20	47.6	10.7
(sucrose)	(n.d.)		(8.89)		(34)	

n.d. = not determined

Table S2. Primer sequences used for RT-PCR amplification of RNA

**Gene**	Forward Primer	Reverse Primer
Cyclophilin A	TCATGTGCCAGGGTGGTGAC	CCATTCAGTCTTGGCAGTGC
CCL2/MCP1	CCTCCTCCACCACCATGCA	CCAGCCGGCAACTGTGA
TNFα	ATCCGCGACGTGGAACTG	ACCGCCTGGAGTTCTGGAA
IL-1β	CTACAGGCTCCGAGATGAACAAC	TCCATTGAGGTGGAGAGCTTTC
Col I	GCTACGCTGTTCTTGCAGTGAT	TCGGTCATGCTCTCTCCAAAC
Col III	TGGCCCTGTTTGCTTTTTATAAG	CGAGATTAAAGCAAGAGGAACACA
FN1	CCCAGACTTATGGTGGCAATTC	TTCGTAATTGGAAGTTGTGCTACAC
EMR1 (F4/80)	TGTCTGACAATTGGGATCTGCCCT	ATACGTTCCGAGAGTGTTGTGGCA
CD68	TTTCTCCAGCTGTTCACCTTGA	CCCGAAGTGTCCCTTGTCA
